# Sustainable Test Methods for Construction Materials and Elements

**DOI:** 10.3390/ma13030606

**Published:** 2020-01-29

**Authors:** Ewa Szewczak, Agnieszka Winkler-Skalna, Lech Czarnecki

**Affiliations:** 1 Group of Testing Laboratories, Instytut Techniki Budowlanej, Filtrowa 1, 00-611 Warsaw, Poland; 2 Thermal Physics, Acoustics and Environment Department, Instytut Techniki Budowlanej, Filtrowa 1, 00-611 Warsaw, Poland; a.winkler-skalna@itb.pl; 3 Scientific Secretary, Instytut Techniki Budowlanej, Filtrowa 1, 00-611 Warsaw, Poland; l.czarnecki@itb.pl

**Keywords:** testing of building materials, test uncertainty, validation of test methods, sustainable test methods

## Abstract

The laboratory testing of the construction materials and elements is a subset of activities inherent in sustainable building materials engineering. Two questions arise regarding test methods used: the relation between test results and material behavior in actual conditions on the one hand, and the variability of results related to uncertainty on the other. The paper presents the analysis of the results and uncertainties of the simple two independent test examples (bond strength and tensile strength) in order to demonstrate discrepancies related to the ambiguous methods of estimating uncertainty and the consequences of using test methods when method suitability for conformity assessment has not been properly verified. Examples are the basis for opening discussion on the test methods development direction, which makes possible to consider them as ‘sustainable’. The authors address the negative impact of the lack of a complete test models taking into account proceeding with an uncertainty on erroneous assessment risks. Adverse effects can be minimized by creating test methods appropriate for the test’s purpose (e.g., initial or routine tests) and handling with uncontrolled uncertainty components. Sustainable test methods should ensure a balance between widely defined tests and evaluation costs and the material’s or building’s safety, reliability, and stability.

## 1. Introduction

**Figure 1:**
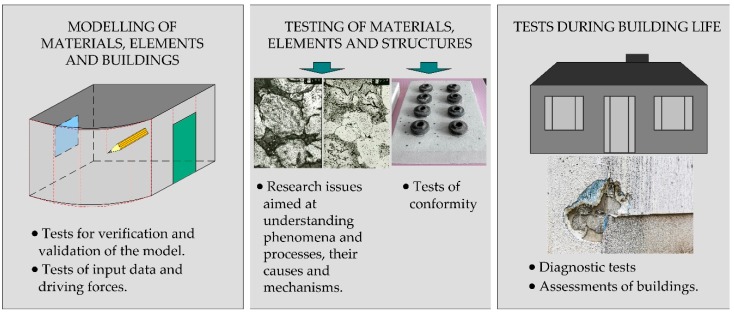
Presence of tests at all stages of the creation and life of buildings.

The construction sector operates a huge number of test methods. Some of them are used to reveal the truth (in a technical sense) about a material, element, or building, while others are simple conformity assessments of products placed on the market. Single laboratory test may give the impression of an irrelevant issue against the complexity of civil engineering. This makes it hard to consider test result uncertainty in this context. However, taking into account all activities related to construction from the moment of its design through all its life cycle, the tests are present at all stages (Figure 1).

The matrix of building science, shown by Czarnecki and van Gembert [1], its multi-faceted nature and complexity in defining building properties implies a colossal number of research and laboratory test methods used in this field including tests of physical and mechanical properties of building materials, chemical, biological, electromagnetic, electric and electronic tests, acoustic and fire tests of buildings and building elements, tests concerning environmental engineering, tests of radiation of building materials, structural tests of materials and others. On the other hand, there is a centuries-old tradition of following the rules of thumb in this area. For many centuries, it gave quite good results. So what is the point of saying about development of precise and modern laboratory test methods? The reason for the need to improve test methods is the accelerated development of materials and technologies, which forces the development of new ways of assessing their suitability. Currently, construction engineering ceased to operate only in the meters and reached the nanoscale [2].

The type of test depends on its purpose. Some of them are individual and specially designed, while others are standardized. If we consider the tests methods according to the scheme in Figure 1, most of them, and above all methods used for validation of models or for the purpose of solving scientific issues are rather individual and developed for a specific need. Examples of such individual tests are on macro-scale the experimental and numerical methods used to study the mechanical behavior of objects at a natural scale (e.g., [3,4]), fire tests of walls or doors [5]. Micro-scale tests are among others scientific methods based on advanced material research, such as X-ray diffraction (XRD), energy dispersive spectroscopy (EDS), scanning electron microscopy (SEM) (e.g., [6,7,8]), and other advanced methods used to understand the mechanisms of phenomena occurring from the macro to the nano-scale. There is also a wide range of individual test and assessment methods used during the service life. Most of them are diagnostic methods such as chemical, ultrasonic, electrical, etc. [9,10] used for building safety inspections. Methods and systems of internal environment testing and sustainability of buildings assessment are rapidly developing (e.g., [11,12,13]). All test methods in all groups shown in Figure 1 are associated with uncertainty and potential dispersion of the results (the dispersion of some results, especially those from individual methods, is not observed because they are performed only once). The issue of uncertainty and precision of the method is, however, particularly important for laboratory tests used to assess the conformity of construction products. These methods should be standardized and ensure an adequate level of compatibility of test results. In this paper, the laboratory tests are generally divided into the research scientific oriented and routine tests of conformity.

**Figure 2:**
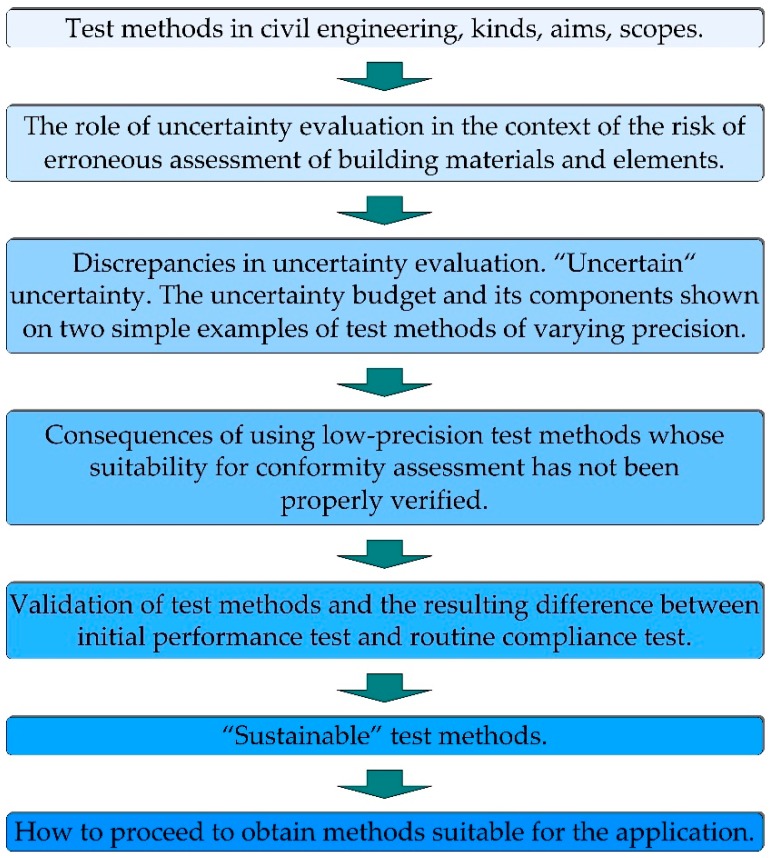
Graphic diagram of the article.

The diagram of this article is shown in Figure 2. The focus is on laboratory tests for conformity assessment because, based on them, decisions are made regarding the possibility of using the material or element in the building. Whenever there is a decision involved, regardless of human activity, there is an uncertainty aspect [14]. Test methods are inseparable from result uncertainty. Realizing the role of uncertainty of the building materials tests results can be easy if we consider that the next, higher assessment level of a complete structure takes into account the sum of partial assessment results, which causes further uncertainty amplification. Thus, the bottom-up procedure is a cluster of sub-models for subsequent assessment models. Result uncertainty may multiply by subsequent uncertainty accumulation at higher assessment levels.

Wherever a decision is related to compliance or non-compliance with specific criteria, this uncertainty involves the risk of committing either a type I error, i.e., rejection of an element which meets the requirements; or a type II error, namely accepting an element which does not meet the requirements. There is also the risk, however, of committing a broadly defined type III error [15] related to asking a wrong question (using the wrong null hypothesis).

Consider the type I risk error in relation to construction materials. This error may incur costs related to the need to create materials or element of a higher performance level than required. This is done in order to avoid erroneous rejection as the result of test uncertainty. The second error type risk is associated with a building’s use-phase and ranges from reduced durability and premature loss of properties through construction disaster extreme cases. In view of these analyses, the type III error risk may mean adoption of incorrect assessment criteria, inadequate testing methods, or incorrect result uncertainty estimation, which may result in the case of wrong material assessment. Therefore, each risk is associated with both: cost and environmental impact (waste, materials with higher content of specific ingredients etc.). In an ‘era of sustainability’ the scientific and research community should strive to minimize such risks at all levels of decisions [16].

Sustainable development in building material engineering is multifaceted and multidimensional [17]. “Sustainable” test method development seems to be part of the progress—such methods help to determine material suitability for specific applications in a credible way, i.e., reducing the risk of threat to life and human health yet avoiding the risk of unnecessary cost generation, excessive use of raw materials, and unnecessary industrial waste generation.

The way uncertainty is taken into account in determining compliance may vary, as outlined in the document of the Joint Committee for Guides in Metrology (JCGM) [18]. When the safe acceptance principle is applied, a test result together with uncertainty must be within the specified limits outlined by permitted tolerance in order to declare a material compliant with the requirements. This material evaluation and material suitability for a specific application in construction involves a typical dilemma: increased cost or increased evaluation safety. This has been discussed in relation to measurements by, e.g., Pendrill [19]. The cost increase encompasses both production costs, including material composition changes in order to be on the ‘safe side’, as well as costs of precise tests. Evaluation error risk reduction can be achieved through measuring device accuracy improvement and increasing the number of samples.

Experience of laboratory test results indicates that, apart from structural materials for the construction industry, uniform and correct rules for uncertainty estimation for other construction material testing are not available. The uncertainty of measurement paradigm evolution has resulted in divergence in uncertainty treatment and estimation ([20,21,22,23,24]). Today’s scientific milieus are inclined to argue that measurement uncertainty is the researcher’s statement on the level of knowledge on a measured quantity. What is worth noting is that uncertainty related issues only pertain to measurements as such. Uncertainty, however, is ignored in scientific discussions in relation to test results. There are few guidance documents [25], which recommend treating tests and measurements alike, but this seems unrealistic, especially in the context of testing building materials. The difference between measurement and test, lies in the fact that in the case of measurements there is a reference value (RV), such as a standard, which is the yardstick to verify trueness. A measurement results should be independent of the measurement method because measurand is physical quantity (like temperature or mass). Uncertainties associated with different measurement methods may be different. A test method is usually specific procedure used to determine the characteristic and there is no reference value for tests. A test quantity definition [26], i.e., a test process model, may be based on several measurements and assessments. A test may offer quantitative results or qualitative results. Quantitative results may not always be a function of the measured values. No uncertainty is attributed to qualitative results at the current knowledge stage. Knowledge obtained from the repeatability and reproducibility of experiments is recommended when estimating the uncertainty of test results. Such experiments may be very expensive and labor-intensive and, thus, should not be the responsibility of a laboratory alone, which has utilized a specific test method. Experiments should be the responsibility of organizations, which develop test methods—these bodies are required to validate methods for intended use. The EA-4/16 guidelines indicate that ‘laboratories cannot in general be expected to initiate scientific research to assess the uncertainties associated with their measurements and tests’ [25].

The ambiguities regarding the basic uncertainty estimation level of test results may result in numerous discrepancies between laboratories. These include model equation discrepancies, considered interactions, estimation of uncertainty components, which stem from result dispersion based on historical data or current results, comparative test results inclusion and different coverage factors. Szewczak and Piekarczuk [27] showed discrepancies in uncertainty estimation and their consequences for final material assessment.

Laboratories differ in the level of their research equipment and staff competencies. We will consider the ideal situation, i.e., laboratories with proven competences (e.g., accredited), having the same level of equipment and eliminating accidental and subjective errors through detailed analysis and participation in proficiency testing. There will still be discrepancies in results due to a number of factors related to the test method. At the same time, even laboratories with the highest level of competence, based on general guidelines for estimating uncertainty, can receive significantly different values of uncertainty, which then affects the assessment of the material tested.

The uncertainties indicated by laboratories may create a wrong impression on the knowledge level of the possible result variability. In some cases, uncertainty is underestimated—this may result in a material obtaining positive assessment results, when such assessment should, in fact, be negative. An overestimated uncertainty used in assessment may, in turn, result in cost increase, which results from the need to improve material properties. It should be noted, however, that both underestimated and overestimated uncertainty values are based on blurred, non-unified prerequisites. Therefore, general principles for considering uncertainty in conformity assessment [18] become irrational if the uncertainty is burdened with even greater uncertainty resulting from the non-uniformity of methods for uncertainty estimation.

The aim of the paper is to show that, when developing test methods, it is not always appropriate to combine the purpose of revealing the truth with the purpose of conformity assessment. Test methods that simulate real conditions may have too many non-controllable parameters that increase the variability of results. This can lead to irrational decisions.

This paper uses simple two independent and contrast test examples, such as bond strength and tensile strength, in order to demonstrate the consequences of using test methods when method suitability for conformity assessment has not been properly verified (i.e., a method has not been properly validated). Uncertainty concept ambiguity in relation to such methods undermines the assessment’s rationality. Although in this article the authors considered only examples of mechanical tests, they believe that the conclusions resulting from simple examples are more universal and form the basis for opening a discussion on the test methods development direction, which makes it possible for the methods to be considered ‘sustainable’.

## 2. Methods

### 2.1. Bond Strength Tests of Adhesives

The tests were carried out on 15 different polymer-cement adhesives, four samples for each adhesive. The adhesives compositions are cement mortars modified with various polymers [28]. In all cases, same-property foamed polystyrene was used as the substrate. The tests were carried out by three different research teams, which used various equipment (reproducibility conditions). They used the pull-off method according to ÖNORM B 6100:1998 10 01 [29]. A layer of polymer-cement adhesive was applied onto the substrate. Once cured in standardized conditions, 50 mm diameter round steel stamps were glued on. A force perpendicular to the surface was applied to the stamps until they pulled off.

Additional tests were also carried out in accordance with the ETAG 004 method [30]—square-shaped stamps of 50 mm were used in this case. The stamp shape impact on the test results is discussed in Section 4.

This can be a basic equation which describes the test model:

(1) $$\sigma =\frac{4F}{\pi D^{2}}$$

where:

*σ*—strength result, MPa
*F*—breaking force, N
*D*—diameter of the stamp in mm.

**Figure 3:**
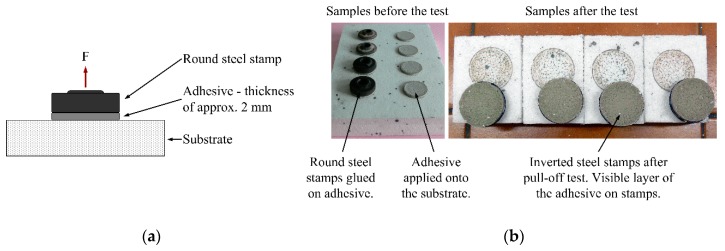
Pull-off test configuration. (**a**) Test scheme; (**b**) photographic documentation of an example test.

It should be noted that the pull-off test is commonly used in different variants, according to different standards and procedures. Configuration of tests used in this article is presented in Figure 3. In addition to adhesives, a pull-off test is used for a wide spectrum of construction materials, including all types of coatings, varnishes, materials for the protection and repair of concrete structures, etc.

### 2.2. Tensile Strength Tests of Plastics

The object of the tensile strength tests were two plastics: ABS-GF and PC. The plastics tensile strength tests example uses the results obtained in laboratory proficiency tests carried out in 69 laboratories. This exercise was organized by the Deutsches Referenzbüro für Lebensmittel-Ringversuche und Referenzmaterialien GmbH (Kempten, Germany). The tests were carried out in accordance with the method described in ISO 527-1/-2 [31] standard. Reproducibility values were published in the organizer’s report [32].

This can be a basic equation, which describes the test model:

(2) $$\sigma =\frac{F}{ab}$$

where:

*σ*—strength result, MPa
*F*—force, N
*a*—sample thickness, in mm
*b*—sample width, in mm.

## 3. Estimation of Uncertainty

### 3.1. Bond Strength Tests and Their Influence on the Results

The general adhesion testing principle is based on stress determination, which results in the tested material detachment from the substrate—the substrate is a laboratory equivalent of the substrate on which the material is actually used. Although the test is intended to simulate the actual conditions of use, it differs in a number of aspects, including:

- The test substrate is usually standardised, thus adhesion to some other substrate in actual conditions may significantly vary.
- The sample is subjected to perpendicular force during the test, whilst the system of forces, e.g., on a façade, may differ in real conditions.
- The material is pulled off with a stamp glued to the sample with epoxy glue, which may affect the sample properties.

Therefore, it is difficult to translate the test result directly into the material’s behavior when installed in the actual building; nevertheless, a test is a uniformed material assessment method and should reflect the differences between particular materials.

The most important factor affecting adhesion results is the adhesive composition. However, in conformity laboratory tests (like in our case) the laboratory only knows the use of the material and the resulting assessment criterion. The criterion must be met regardless of the composition.

The factors which can affect the test results (their uncertainty) can be divided into three groups:

1. Measurements which directly affect the result. According to Equation (1), these are the force measurement at pull-off and the stamp diameter attached to the sample. These measurement uncertainties can be determined in two ways—either in relation to the calibration certificates or in relation to the confirmation of compliance with conditions specified in the standards as uncertainty. For example, if the force ought to be measured with a Class 1 device, it means that the total uncertainty of measurement should not exceed 1% of the measured value.
2. Measurements with uncertainties which do not affect the result according to a known relationship; however, they are measured in order to maintain compliance with the tolerance limits given in the standard: ambient temperature, air humidity measurement, force application speed measurement.
3. Interactions whose level is not measured: force application direction (some non-perpendicular components of stress) which should be controlled by the construction of the measurement device, but there are always some imperfections, the substrate and stamp surface difference, the epoxy glue chemical and physical influence on the adhesive, repeatability, and reproducibility of sample preparation (the sample preparation process for testing is multi-stage and involves a number of interactions, such as layer thickness, clamping force, conditioning variability, and others that may affect the test result), the material and sample heterogeneity.

It is noteworthy that in the case of all tensile strength tests there are usually some non-perpendicular components of stress. In some cases, first of all, related to scientific oriented research, emerging non-perpendicular components are the subject of analysis aimed at understanding material behavior and enriching knowledge about the material, such as in rock-engineering performance where visible discontinuities created by nature and adjacent areas (on a mm-scale) differing significantly in mechanical properties in tested material cause variability of tangent modules [33]. In the case of bond strength conformity tests, however, they will constitute an undesirable factor resulting from the imperfections of the measurement system and preparation of the sample. Ultimately, it reveals itself in a dispersion of results along with other uncontrolled factors. In this example, one can see a significant difference between compliance and scientific tests. The same influencing factor is, in one case, a source of information, and in the other, an obstacle to the precise assessment.

The bond strength test, like most tests used to assess construction materials, is of a destructive nature; thus, it is not possible to repeat the action sequence on the same sample. This situation makes it impossible to isolate the impact of individual components from groups 2 and 3 on the result. Thus, from a practical point of view, a laboratory which performs a specific test obtains a limited test result pool (3 to 5 results) and the knowledge of the lab encompasses the device measurement characteristics and the final dispersion of the results.

If the tolerance limits are given in the description of the test method, it is assumed by default that observance of these limits ensures that group 2 interactions have a negligible impact on the test results. The variations related to material heterogeneity, sample preparation, and the test itself are manifested in the final dispersion of the results. Given the fact that a laboratory normally tests 3–5 samples, the listed components must be considered in conjunction.

Thus, in this situation, we can determine the components determined by measurement of force and diameter. These components can be included in the model equation. The rest of the components are contained in the dispersion of results; however, their impact remains unknown. It would be necessary to perform numerous inter-laboratory experiments in order to separate components related to the test from the components which result from the tested material or samples heterogeneity.

Such experiments are justified during the method validation period but few specifications indicate the method precision (standard deviation of repeatability and reproducibility), which could be treated as the uncertainty component.

#### 3.1.1. Precision of Bond Strength Tests

Documents [29,30], which describe and recommend the use of bond strength tests, include no data on test precision. Numerous outcomes based on both repeatability and reproducibility tests indicate that a very large dispersion of results is characteristic for bond strength tests.

Table 1 shows the bond strength test results (average values of four lab tests for each adhesive in each laboratory) in the three research teams. The tests used 15 different polymer-cement adhesive samples of 0.02 to 0.11 MPa adhesion range. The table also includes the result variability for individual adhesives in individual laboratories, as well as variations resulting from repeatability and reproducibility calculated in accordance with the ISO 5725 [34] based on all laboratories’ results.

All variations in Table 1 are expressed as a *v* coefficient of variation for better presentation:

(3) $$v=\frac{s}{\bar{\sigma }}\;100\%$$

where:

*s*—standard deviation of repeatability or reproducibility
$\bar{\sigma }$—mean value of bond strength in the result set with standard deviation calculated.

The data was subjected to the Grubbs test for outliers within the result set for a given adhesive. The test showed no outliers. The individual standard deviations for the three laboratories and each adhesive were subjected to the Cochran test. A questionable standard deviation value of 0.024 MPa (*ν* = 55.6%) was obtained for the ‘e’ sample and Laboratory 2. One standard deviation outlier of 0.021 MPa (*ν* = 33.4%) was obtained for sample ‘n’ and Laboratory 1. In each case, an increased standard deviation was recorded in a different research team; thus, the questionable and outlier variance results were not rejected in the overall variance of repeatability and reproducibility calculations.

There is no statistically significant correlation between the standard deviation of repeatability and reproducibility values and the adhesion force value (respectively *r* = 0.13 and *r* = 0.38 for the critical value with the *α* = 0.05 coefficient *r*_cr_ = 0.514). There is no statistically significant correlation between intra-laboratory and inter-laboratory standard deviation either (*r* = 0.16) or between standard deviations in particular laboratories (*r* lies between 0.18–0.28).

The dispersion values are random and do not indicate that any of the laboratories involved in the experiment has demonstrated standard deviations, which were too high or too low. Minimum and maximum v values for repeatability are 9% and 42%, and for reproducibility 11% and 45% respectively. These values are very high and, thus, indicate that the test method is of low precision. Given that the adhesive assessment criterion is: *σ* > 0.08 MPa, the result variability may suggest that this adhesive assessment test method involves a high risk.

#### 3.1.2. Estimation of Bond Strength Results Uncertainty

The uncertainty estimation was carried out with the use of methods recommended in JCGM documents [20,21] and EA [25].

Considering the interaction impact assessment on the test results, the model equation, which makes it possible to determine the individual components influence on the total uncertainty of the adhesion test results, is

(4) $$\sigma =\frac{4F}{\pi D^{2}}+A_{\sigma }$$

where:

*Aσ*—unknown value interaction impacts, which contribute to the result dispersion (includes material heterogeneity). The value *Aσ* = 0—the error value which could be corrected is unknown; however, *Aσ* uncertainty contributes to *σ* uncertainty. This uncertainty is revealed in a random dispersion of results expressed as the standard deviation of the σ value.

Other elements as in Equation (1).

The total value of standard uncertainty estimation *σ* is usually based on equation:

(5) $$u_{c}^{2}\left(y\right)=\sum {\left(\frac{\partial f}{\partial x_{i}}\right)}^{2}u^{2}\left(x_{i}\right)$$

hence

(6) $$u_{\sigma }^{2}={\left(\frac{\partial \sigma }{\partial D}\right)}^{2}u_{D}^{2}+{\left(\frac{\partial \sigma }{\partial F}\right)}^{2}u_{F}^{2}+{\left(\frac{\partial \sigma }{\partial A_{\sigma }}\right)}^{2}u_{A_{\sigma }}^{2}$$

where:

*u_D_*—standard uncertainty of *D*
*u_F_*—standard uncertainty of *F*
$u_{A_{\sigma }}$—dispersion of *σ* results.

Expanded uncertainty is

(7) $$U_{\sigma }=ku_{\sigma }^{2}$$

where *k* is the coverage factor corresponding to 95 percent coverage interval.

Owing to the fact that there are no uniform uncertainty estimation principles and that, according to recommendations, uncertainty estimation should be based on the state of knowledge, laboratories may make different assumptions about the value of *u_D_*, *u_F_*, *a*, and *k*, although they use the same equations as the starting point. For example, when estimating type B uncertainty related to force values, either measurement tolerances or specific uncertainty from the calibration certificate might be taken into consideration. Although $u_{A_{\sigma }}$ laboratories mostly rely on historical data of the repeatability test on a larger number of samples in a given laboratory, the current series standard deviation might be used in many instances. Reproducibility standard deviation obtained from inter-laboratory experiments should be used, as recommended [25]. This, however, is a rare case.

*k* = 2 is sometimes taken as the coverage factor, although this assumption is not always justified (approximate normal distribution value corresponding with 95 percent coverage interval). *k* obtained from Student’s *t*-distribution is also used, based on the effective number of degrees of freedom calculated from the Welch—Satterthwaite equation. The current approach outlined in the JCGM document [21] consists in the probability density distribution (PDF) propagation rather than the uncertainty propagation—this may also produce different uncertainty results.

Table 2 presents some examples of how uncertainty components can be estimated and how can be used with the model equation presented herein. All of them have been created by the authors in accordance with the possibilities presented in the JCGM documents [20,21].

**Figure 4:**
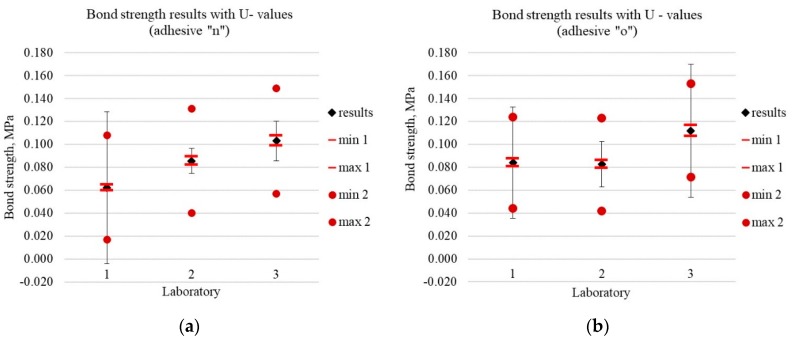
Bond strength results with U value for three laboratories (1–3) and two materials (**a**) adhesive ‘n‘; (**b**) adhesive ‘o’. Black error bars are based on actual standard deviation (approach II). Points min 1 and max 1 are 95% coverage intervals obtained in approach I (only B-uncertainty of *F* and *D v*alues), points min 2 and max 2 are 95% coverage intervals obtained by the Monte Carlo method in approach IV, where reproducibility standard deviation obtained from inter-laboratory comparisons was used.

Figure 4a,b show the test results and coverage intervals resulting from the use of different approaches towards uncertainty estimation (I, II, and IV). The uncertainties estimated with the use of different approaches differ significantly, which seems particularly important. The detailed results are shown in Table 3.

The result differences in particular laboratories when testing the same adhesive are significant enough to affect the final conformity assessment against the requirements. The uncertainty value differences obtained by different laboratories using different approaches, and uncertainty values which exceed the test result value (e.g., approaches II and III for Lab 1, adhesive ‘n’) weaken the significance of such “uncertain” uncertainty when assessing the risk of incorrect assessment.

ISO 527 [31] based tests examine the tensile stress of plastics. Unlike bond strength testing, application conditions simulation is not the test objective; rather, the test results describe the material properties. A designer outlines the material conditions and, thus, determines material suitability for specific construction use.

### 3.2. Tests of Tensile Strength and Their Influence on the Results

The factors which can affect the test results can be divided into three groups, as with bond strength:

1. Measurements which directly affect the result. According to Equation (2), these are the force, thickness and width of the sample measurements.
2. Measurements with uncertainties which do not affect the result according to a known relationship; however, they are measured: ambient temperature, sample geometry, and stress increase pace.
3. Interactions whose level is not measured yet affects the result, include, but are not limited to, the jaw design which may result in excessive strain exerted on the sample or may cause the sample to slip, heterogeneous sample thickness, and material heterogeneity. The method assumes perpendicular stress, however, the design of the equipment and inaccurate clamping of the sample may imply the formation of tangential and torsional components that may affect the result.

#### 3.2.1. Precision of Tensile Strength Tests

Tensile strength test precision is described in the ISO 527 standard applicable for various types of plastics. For PC repeatability and reproducibility standard deviations: sr = 0.18 MPa, sR = 0.89 MPa, for ABS sr = 0.18 MPa, sR = 1.93 MPa respectively.

As part of the laboratory proficiency testing organized by the Deutsches Referenzbüro für Lebensmittel-Ringversuche und Referenzmaterialien GmbH (DRRR), the standard reproducibility deviation values were determined: for PC sr = 0.26, sR = 0.73 MPa, for ABS-GF sr = 0.51 MPa, sR = 1.35 MPa. DRRR inter-laboratory studies involved 69 laboratories, thus statistical data is very extensive.

The experiment results make it possible to conclude that, in contrast to the bond strength tests, according to ISO 527-1,2 [31] a random dispersion of results in the tensile strength test expressed as the reproducibility standard deviation is relatively small.

Variation coefficient *v* defined by Equation (3)—PC and ABS-GF respectively—repeatability: 0.4% and 0.9%, and reproducibility: 1.3 and 2.3%. Compared to the v value determined in bond strength tests, these values are significantly lower.

#### 3.2.2. Estimation of Tensile Strength Results Uncertainty

The model equation which makes it possible to determine the individual components influence on the total uncertainty of tensile strength test results can be presented in the same way as was the case with bond strength tests (Equation (4)):

(8) $$\sigma =\frac{F}{ab}+A_{\sigma }$$

where:

*Aσ*—unknown value interaction impacts which contribute to the results dispersion (includes material heterogeneity). Other elements as in Equation (2).

Standard uncertainty of σ is:

(9) $$u_{\sigma }^{2}={\left(\frac{\partial \sigma }{\partial a}\right)}^{2}u_{a}^{2}+{\left(\frac{\partial \sigma }{\partial b}\right)}^{2}u_{b}^{2}+{\left(\frac{\partial \sigma }{\partial F}\right)}^{2}u_{F}^{2}+{\left(\frac{\partial \sigma }{\partial A_{\sigma }}\right)}^{2}u_{A_{\sigma }}^{2}$$

where:

*u_a,b,F_*—standard uncertainty of *a*, *b*, *F*
$u_{A_{\sigma }}$—dispersion of *σ* results.

Uncertainties were estimated based on ISO 527 standard assumptions, i.e., force measurement with the use of a class 1 device, requirements for the sample shape: a = 10.0 ± 0.2 mm, b = 4.0 ± 0.2 mm.

**Figure 5:**
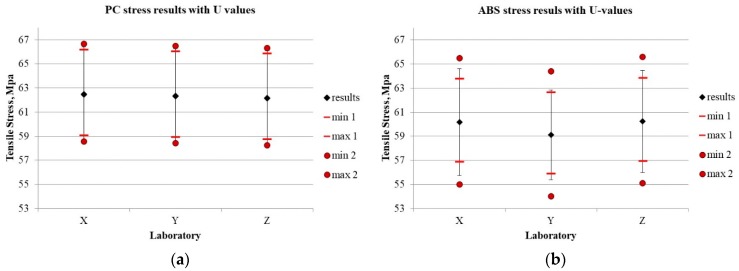
Tensile strength results with U value for three laboratories (X, Y, Z) and two materials (**a**) PC, (**b**) ABS. Black error bars are based on actual standard deviation (approach II). Points min 1 and max 1 are 95% coverage intervals obtained in approach I (only B-uncertainty of *F* and *D* values), points min 2 and max 2 are 95% coverage intervals obtained by the Monte Carlo method in approach IV, where reproducibility standard deviation is obtained from ISO 527-2 standard.

Figure 5a,b show the test results and coverage intervals which result from the use of different approaches towards uncertainty estimation (I, II, and IV). The different approaches to uncertainty estimation differ slightly, especially when compared to the differences in the uncertainty values in the bond strength test. Therefore, the estimated uncertainties appear to provide the basis to claim that the risk of incorrect assessment can be determined in a credible manner. The data referring to the method precision given in the standard may be the basis for a uniform uncertainty estimation.

## 4. Discussion

### 4.1. Comparison of Methods in the Context of Uncertainty

The comparison of similar model-based test methods (Equations (1) and (2)) indicates a large disproportion in reliability of the values’ uncertainty determination.

What is important is that the majority of uncertainty components in tests in accordance with ISO 527 [31] can be controlled, as opposed to the bond strength test. This can be observed by comparing Figure 4 and Figure 5. Points min1–max1 indicate the uncertainty extent, which results from the accuracy of the measuring devices used (measurement of sample strength and geometry). This is the measurement uncertainty and is epistemic in its very nature (according to the Walker matrix [14]). The measurement uncertainties described in the test method requirements outlined by the author of the method are normally used (as is the case with this paper). This is the easiest way and provides the most consistent uncertainty data. In some cases, this uncertainty can be overestimated, as the actual measuring device used in a given laboratory may be more accurate than the one referred to in the standard. Thus, more accurate devices can also be used in order to reduce uncertainty.

**Figure 6:**
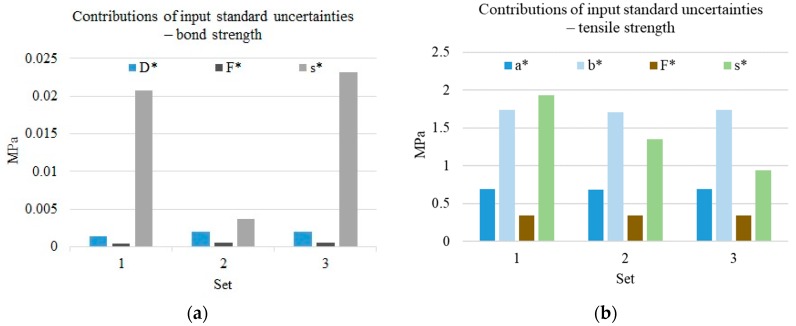
Contributions of input standard uncertainties: (**a**) *D*, *F* and *A_σ_* contributions to bond strength combined uncertainty. Sets of bars 1 and 2 have been obtained for two laboratories with different actual standard deviation (II approach), set 3 was obtained for laboratory 2 which used reproducibility standard deviation. D* is the $\frac{\partial \sigma }{\partial D}u_{D},$ F* is $\frac{\partial \sigma }{\partial F}u_{FB}$ and A* is $\frac{\partial \sigma }{\partial A\sigma_{}}u_{A\sigma }$. (**b**) *a*, *b*, *F*, and *A_σ_* contributions to ABS tensile strength combined uncertainty. Set 1 is based on reproducibility standard deviation from ISO 527-2; set 2 is based on reproducibility standard deviation obtained in DRRR inter-laboratory experiment; set 3 is the actual standard deviation of an exemplary laboratory. a* is the $\frac{\partial \sigma }{\partial a}u_{a},$ b* is the $\frac{\partial \sigma }{\partial b}u_{b},$ F* is $\frac{\partial \sigma }{\partial F}u_{FB},$ and s* is $\frac{\partial \sigma }{\partial A\sigma }u_{A\sigma }$.

The component *Aσ* presented in this paper is the uncertainty of aleatory nature associated with external and internal interactions (the test model inherent), which remain outside the scope of control. If introduced, this component determines the limits of the min 2 and max 2 interval in Figure 4 and Figure 5. The impact of individual components on uncertainty is shown in Figure 6. While the uncontrolled component ($s^{*}=\frac{\partial \sigma }{\partial A\sigma }u_{A\sigma }$) in the test carried out according to ISO 527 [31] is of the same order as the other components, in the case of the bond strength test, the uncontrolled component is many times higher, although there are also some results with a randomly smaller dispersion.

The presence of large-scale uncontrolled components of variable values in bond strength tests may undermine confidence in both the test results and the reported uncertainty. The results’ confidence is expressed and demonstrated by the error risk assessment. Type I errors are directly related to the production costs increase. Type II errors may affect the material users‘ safety. In the above case, there may be a high risk of type III errors related to the test model, which sets the assessment criteria incorrectly.

Compared to the bond strength test, the tensile stress test method according to ISO 527 demonstrates much better precision (repeatability and reproducibility). Uncertainty components that result from uncontrolled interactions contribute less towards total uncertainty. Thus, it can be concluded that the tested value definition in the tensile strength minimizes uncontrolled interactions, in contrast to this definition of bond strength. This is related to the fact that the bond strength test is more complex, and involves more operations and more factors affecting variability.

### 4.2. Effects Related to Material Assessment

**Figure 7:**
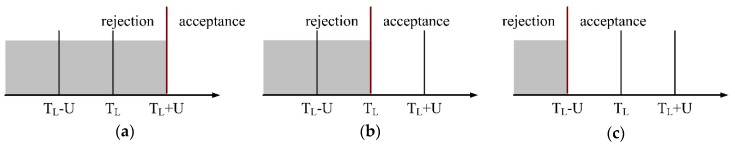
Methods of conformity assessment with the use of uncertainty values, based on the example of the lower tolerance limit *T_L_*. (**a**)—guarded acceptance; (**b**)—simple acceptance; (**c**)—guarded rejection. *U*—expanded uncertainty value.

According to current JCGM guidelines [18], there are three main ways to account for uncertainty in conformity assessment test results with the tolerance limits delineated in the material requirement:

(a) Guarded acceptance (as shown in Figure 7, a lower acceptance limit *A_L_* = *T_L_*+ *U*, where *T_L_*—lower tolerance limit and *U*—expanded uncertainty)
(b) Simple acceptance (a lower acceptance limit *A_L_* = *T_L_*)
(c) Guarded rejection (lower acceptance limit *A_L_* = *T_L_* − *U*)

In the case of applying the principle of guarded rejection, sometimes used in factory production quality assessment, the lower acceptance limit might be (0.08 − *U*) (MPa). For uncertainty estimated using approach II by laboratories 1, 2, and 3 it is: 0.014, 0.069, and 0.063 MPa respectively (adhesive “n”). Note that the first value is more than five times lower than the tolerance limit (0.08 MPa).

If guarded acceptance is used, a sample tested by any of 1–3 laboratory which estimates uncertainty according to Approach III, would have to find an adhesion of 0.15 MPa and, thus, almost double the required value (0.08 MPa). This would involve a significant change in the adhesive composition—in polymer cement adhesives this would most likely result in a significant increase of polymer amount in relation to cement. Table 4 presents decisions about rejection or acceptance of adhesive used as insulation material bonding to a wall, depending on the laboratory approach used for uncertainty evaluation and the way of using uncertainty in the assessment.

### 4.3. Difference between Initial Performance Tests and Routine Compliance Tests on the Background of Validation of Test Methods

Validation before method implementation is normally used in order to confirm the test method suitability for a particular application. The first validation stage is setting the requirements a test method should meet. If the method is supposed to provide the material assessment against established criteria, e.g., tolerance limits (*T_L_*—lower limit, *T_U_*—upper limit, tolerance range width *T* = *T_U_* − *T_L_* ) assigned to a given material class, this parameter is described as

(10) $$\alpha =\frac{U}{T}$$

the measurement accuracy factor. The factor should be of a certain value in order to make it possible to evaluate whether the criteria based on the results of the measurement are met. Metrology, in a customary manner, assumes that this factor is not greater than 0.1 for greater responsibility measurements and 0.2 for measurements of smaller responsibility. In the bond strength test, where *T* = 0.08 MPa, the uncertainty should be around 0.008 MPa (or 0.016 MPa). Uncertainty at the level of 0.1*T* would only be achievable when approach I is used, i.e., the budget includes component uncertainties arising only from requirements regarding the accuracy of measuring devices. The uncertainty of 0.2*T* is sometimes achievable when the dispersion of results budget resulting from repeatability is included. Given the large differences in the standard deviations obtained in the tests carried out by different laboratories for different materials; however, the uncertainty estimated seems unreliable.

The examples above clearly indicate the test method’s deficiencies when used for material evaluation; these deficits are manifested by result differences. From a mathematical point of view, a rational assessment becomes impossible. In addition, assessment is unreliable because there are no unified rules for uncertainty estimation and no arbitrary assessment principles adopted for all parties (Table 4).

As already mentioned, the reasons for the test method deficiencies are mainly related to the large number of interactions which impact the result. This stems from the attempts to develop a method similar to natural conditions.

The substrate to which the tested adhesive is applied in order to assess adhesion is one of these interactions. The following cases can be considered:

1. The test is to be carried out with the use of the substrate which the adhesive will be applied to in regular conditions of use. This solution may prove very costly for adhesives which can be used for a variety of materials.
2. The test is to be carried out with the use of a reference substrate (the same for all laboratories which use a particular method, irrespective of subsequent use of the material). This increases the method precision; however, it makes the method-based test results diverge from the actual conditions of use, which in turn increases the type III risk associated with the use of an incorrect assessment criterion.

In the test methods cited in this paper, the second solution is used. This means that the result variability related to the impact of the substrate is limited. The assessment of how the adhesive will behave in practical application on the actual substrate; however, remains outside the scope of this model.

If R denotes an abstract difference value between the material performance in actual conditions (*Yu*) and the test result (*Yt*), the use of the actual substrate for tests results in *R* reduction and, thus, increases the testing costs (many substrates for different material applications). It may also increase uncertainty. This stems from the fact that there is no specification regarding the substrate type which may cause discrepancies between laboratories’ choice regarding test substrate types which approximate the substrate of intended use.

The shape of the adhesive layer on the substrate is another interaction example where result variability has been limited. For instance, ETAG 004 [30] based tests require the use of a 50 mm side square stamp in the adhesive removal test. For adhesive property tests carried out according to previous recommendations in Poland, the method described in point 2 was used—a round stamp of 50 mm diameter. Theoretically, this should not affect adhesion which, by definition, is the force related to a surface unit. The Building Research Institute’s practical tests, however, demonstrated different results for the same adhesives and substrates (Table 5).

The surprisingly large average value differences shown in Table 5 may also be attributed to the poor repeatability of the method, as discussed in Section 3.1.1.

The test method requirements impose specific stamp dimensions and shapes. The adhesive layer at the actual construction site is applied to several places on a foamed polystyrene board. The adhesive layer shapes and surfaces are uncontrollable (or controlled within wide tolerances, as the instructions are not very precise and are related to workers’ practical experience). Considering the above variable, there is no rational possibility to establish the relation between material performance in actual conditions (*Y_U_*) and test results (*Y_t_*). In the case of the stamp, the imposed shape may only reduce the dispersion of test results.

It is apparent that whenever the test method does not perfectly duplicate the actual conditions of use, the variability increase related to the test method itself also increases the risk, when the test results are transferred to the conditions of use. In-test interactions and actual conditions interactions do not compensate, because they are random. Therefore, the final properties variation in actual conditions of use must include the test result variability resulting from the test interactions, as well as the variability resulting from the actual conditions of use. This can be expressed symbolically with equation:

(11) $$D^{2}\left(R\right)=D^{2}\left(Y_{u}\right)+D^{2}\left(Y_{t}\right)$$

where *D*^2^ means variance.

Hence, the greater the variability of the test method itself, the greater the variability when the method is transposed to the actual conditions of use and, thus, the greater is the risk of erroneous assessment.

It should be noted, however, that both *Y_u_* and *U*(*Y_u_*) are virtually unknowable and, thus, can be only the subject of modeling, burdened with further uncertainties of the model and its implementation. As for the test result and its uncertainty, the examples presented in this paper also demonstrate the important property of tests methods consisting in the ‘unknowability’ of ‘true’ results. The issue of unknowability in relation to measurements and measurement uncertainty are extensively discussed by Grégis [36]. Unlike measurement methods, the level of unknowability of test results is even greater—there is no ‘true’ value. There is no reference value (RV) either. If one is capable of imagining a ‘real’ length value (although this ‘real’ is still in the sphere of abstraction) as there are reference values to which the measurement results’ trueness can be referred to, it is very difficult to imagine RV for test methods, especially for those used for construction materials. The bond strength tests seem to be one of the simpler examples where it is not completely unimaginable to have a test standard (although it would be destroyed with each test). It would be extremely difficult, however, to create a test standard of a curtain wall resistance to heavy body impact or of a fire door resistance. Measurements are included in each of the tests mentioned above (e.g., force measurement, the weight of the impact body, temperature), which may have RVs; however, these are only components whose influence on the final test result is unknown in most cases due to the very large number of other interactions.

Considering the above, the unknowability of properties in the actual conditions of use (*Y_u_*), the test result (*Y_t_*) and the variance of these values undermine the possibility of material assessment by means of laboratory tests. There are laboratory tests, however, which are the basis for material assessment. Material property criteria for actual use are, thus, developed. This is the domain of reality modeling. Attempts to achieve the best convergence between the model and reality are universal. This should also apply to test methods. The actual purpose of the test, however, should be considered.

**Figure 8:**
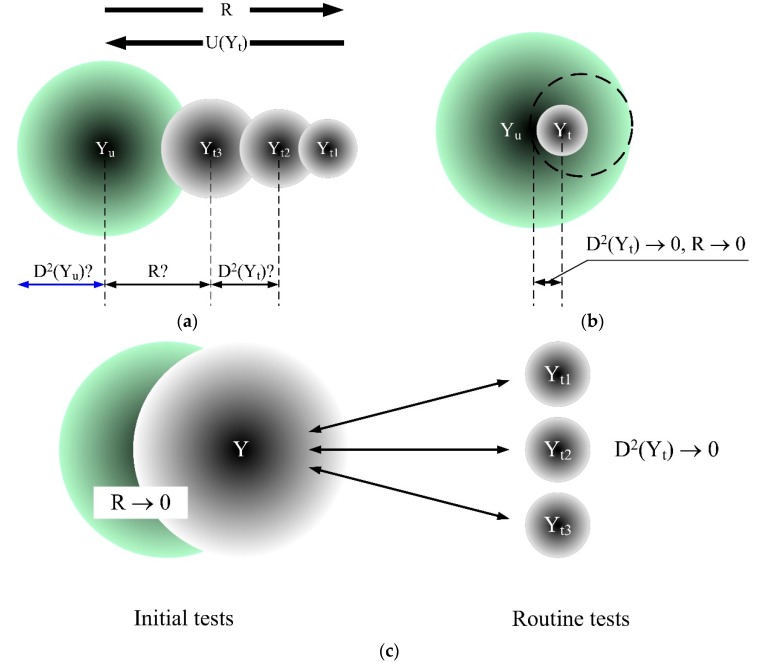
Illustration of the discrepancy between the material’s properties in actual conditions of use (*Y_u_*) and the test result (*Y_t_*), *R* = *Y_u_* − *Y_t_*. *D*^2^(*Y_t_*) —variability of test results. *D*^2^(*Y_u_*)—symbolic variability of actual conditions. (**a**) Test results uncertainty reduction which results in a difference increase when related to actual conditions. (**b**) Simultaneous *R* and *D*^2^(*Y_t_*) minimization. (**c**) Tests modeling depending on the purpose.

As already mentioned, bringing the test model closer to actual conditions reduces the *R* value (Figure 8a); however, it may increase test result uncertainty with the increased number of interactions that impact on the result. The ideal situation we seem to be striving for is both small test result uncertainty and maximum approximation to actual conditions. As shown in Figure 8b, this is practically impossible, because as *R* and *D*^2^(*Y_t_*) are concurrently reduced, the point is reached where the common part of fields (*Y_u_*, *D*^2^(*Y_u_*)) and (*Y_t_*_,_ *D*^2^(*Y_t_*)) is also reduced.

Thus, this optimization issue can only be an objective function (Figure 8c). If the comparability of the test results of individual materials carried out by different laboratories is the objective, the minimization attempt should characterise the *D*^2^(*Y_t_*) test method. If the method is of a cognitive nature, where reality modeling and ‘revealing the truth’ is the objective [37], it is necessary to minimize the *R* value. Following this logic, the initial material test in the design phase should use a different method from the routine test method used in the assessment and verification of constancy of performance (AVCP) [38] or any conformity assessment with the standards.

The initial material and elements test should be a model of reality and should simulate actual conditions. The individual application of such methods reduces uncertainty mainly to aspects related to modeling. Verification and validation of models is a slightly different issue than the validation of simple, repeatable test methods. The uncertainty inclusion in model verification is described in the publications of Scheiber et al. [39] and Oberkampf et al. [40].

A routine test should only be a model of a particular aspect of reality simple enough to deliver predictable results. In the conformity assessment tests in AVCP processes, the test development phase should include the incorrect assessment risk estimation resulting from the simplified test model, as well as the incorrect assessment risk resulting from the method precision. The rational level of required uncertainty *U* determination should be the responsibility of the organization which develops the method.

The test method principles should therefore be formulated as:

(12) $$u_{c}^{2}\left(y\right)=\sum {\left(\frac{\partial f}{\partial x_{i}}\right)}^{2}u^{2}\left(x_{i}\right)+s^{2}<{\left(\alpha T\right)}^{2}$$

where:

*α*—targeted accuracy factor related to standard uncertainty and tolerance limits
*T*—material properties tolerance range
*f*—function which describes the measurement model.

All components related to the test devices used and other factors that are controlled can be included.

(13) $$u_{k}^{2}=\sum {\left(\frac{\partial f}{\partial x_{i}}\right)}^{2}u^{2}\left(x_{i}\right)$$

The method suitability condition can, therefore, be represented by equation

(14) $$s^{2}<{\left(\alpha T\right)}^{2}-u_{k}^{2}$$

which would limit the dispersion of results not attributed to the specific, known components of uncertainty described by Equation (13).

This validation method can be relevant for the organization which develops the method in order to ensure that the reproducibility variance in the final validation experiment does not exceed value $s^{2}$. This is also important for laboratories which would have to show a sufficiently small dispersion of results with the use of the particular method. Therefore, in specific tests the laboratories would reject results whose deviations would demonstrate a higher variance, as high probability assumption means that the variance is related to material properties rather than the test. As can be seen from the above, material assessment criteria on the basis of tests should not only relate to the values but also to their variance.

The presented approach to improving conformity test models by supplementing them with conditions regarding the required variability of results offers benefits. The ambiguities associated with uncertainty estimation are eliminated. Instead of presenting uncertainty associated with a high risk of error, as has been shown, the laboratories would have to demonstrate the test method’s individual requirements fulfilment. This, in consequence, would lead to uniform uncertainty levels and uniform material assessments in different assessment bodies.

### 4.4. Sustainable Test Methods

Test method development, both the initial material assessment in the design phase as well as assessment routine, is based on the balance between economy (research costs) and safety (error risk). Both aspects relate to energy, raw materials consumption, waste generation, and environmental and social costs in the event of an accident.

Sustainable test methods ensure a balance between widely defined tests and evaluation costs and the material’s or building’s safety, reliability and stability.

Considering the general risk assessment principle:

*risk* = (*probability of undesirable outcome*) × (*effects of undesirable outcome*) (15)

where effects can be broadly defined. One should consider probability and effects (economic, environmental, social, and other costs) associated with unnecessary spending on the development of a well-validated assessment method: the increase of the number of samples and equipment accuracy on the one hand and risk failure as a result of incorrect assessment (product of the failure probability and failure effects) on the other. Such comparison differs among material types and applications. This explains the high expenditure and great attention paid to construction materials load capacity assessment methods and the limited attention paid to test methods of finishing materials. In the first case, the failure effects impact human safety.

If we consider the effects associated with building durability, raw materials, energy consumption, and repair costs, however, well-justified and validated testing and assessment methods should also be applied from a sustainability point of view in relation to materials which are less important.

Test method optimization to ensure their ‘sustainability’, as shown in Figure 8, can be obtained by separating test models designed to learn about material or element behavior in actual conditions from method precision problems. Thus, test methods should be divided into initial and routine.

### 4.5. Final Remarks

A model only approximates reality. There is a common goal, however, to bring a model as close to reality as possible. This is also science’s objective. Given what Czarnecki et al. [37] noted, a picture of reality is dependent on the reference system used. Construction materials engineering uses reality models and may use different reference systems. The latter should be appropriate for the purpose the model has been created. A test method used to assess future material behavior in actual conditions is one of the sub-models, which constitute the final model of material properties. Real conditions, however, are associated with a full range of influences. Some of the interactions are controlled by the model, others may be partially known, yet not included in the model structure. This knowledge has not yet been researched. There is also a group of interactions outside the research scope when the model was created. The creation of test methods, which simulate the actual conditions of use, often leads to the following situation: too many aspects are outside the study model’s control in real conditions. An attempt to clarify these aspects increases method precision but, at the same time, makes the method actually diverge from real conditions. This can be optimized by the development of a test method set used depending on the purpose: as an initial test when designing a material or as a routine test when assessing its legal conformity.

Considering the risks and the costs associated with the use of certain types of assessment methods based on tests, increased uncertainty of test results used for assessment increases the likelihood of making a wrong decision, and results in more adverse effects associated with that wrong decision. The associated tipping points are as follows:

- a non-compliant material or element is put on the market, which implies broadly defined costs for the producer related to the batch’s withdrawal
- installation of non-compliant materials—this may result in failures, repair mitigation costs, as well as immeasurable costs of impact on health and safety.

Considering erroneous assessments, type III error risk cannot be ignored, as it has been demonstrated in the paper, because of non-validated test methods, lack of unambiguous findings regarding uncertainty estimation in relation to given tests, and lack of uniform rules of material assessment which account for uncertainty. All adverse effects of erroneous assessments also impact the environment through raw materials, energy consumption, and waste generation. Inadequate construction material durability falls short of future building users’ expectations and results in higher future renovations costs.

The bond strength study example shows that inappropriate test methods, which do not ensure adequate results reliability, can increase the assessment risk area so much that the assessment becomes irrational. It should also be noted that the simple examples shown in this paper constitute only a fraction of the uncertainty problem. The study considers methods, which produce quantitative results. There is a plethora of methods, however, which produce results on a nominal scale or offer qualitative results (such as ageing methods for durability assessment, resistance tests, etc.) where uncertainty, and hence the risk of incorrect assessment, is extremely difficult to estimate.

Given the importance of sustainability in all areas of human activity, it also seems reasonable to refer to this aspect in test methods in the construction sector. As shown in the paper, a sustainable test method is a method which does not generate undesirable economic, environmental and social effects because of incorrect assessments. In order to consider a method as sustainable, it must be first of all fit for the intended purpose. Whenever necessary (initial tests of innovative products), it ought to present the actual conditions of use. In other cases (methods used routinely in assessments), the method should offer sufficiently good precision for the assessments of materials and elements results to be unambiguous. The development of methods and understanding the relationship between initial and routine assessment and actual conditions of use are scientific issues.

The applicable and reasonable basis for the uncertainty level is an important aspect of test methods, primarily routine ones. This uncertainty level should be the same for all laboratories involved in AVCP and any conformity assessment, established on assessment criteria and methods which take previously established uncertainty into account, and available for all stakeholders. Therefore, the sustainable test methods model should also include the uncertainty estimation procedure, which ensures a uniformed code of conduct for all those who use the method. Universal methods of estimating uncertainty can be interpreted in many ways, as have been shown in point 3, therefore, the only solution is to specify a specific and strict uncertainty assessment procedure addressed to a specific method. This procedure should be developed by the institution that develops the test method during its validation (which is currently very rare) and used by the method users. The level of controlled and uncontrolled interactions should be determined. Controlled interactions should be presented as requirements (e.g., related to the metrological properties of the equipment, the test environment, etc.). Uncontrolled interactions and its acceptable levels within a single test series under repeatability conditions, as well as within reproducibility should be evaluated during the validation experiment’s. Laboratories, which use the test method, should be able to prove their capacity to carry out the test by showing a dispersion of results not greater than that given in the procedure. Then, in the case of results with a larger dispersion, a material can be rejected.

In the construction sector, there is a number of test methods aimed at construction product performance assessment; however, there is a limited number of methods which meet the conditions described above, although the method development costs (largely the costs of validation experiments) may be incomparably smaller compared to the adverse effects resulting from incorrect assessment. Development in this area should include better characterization (model improvement) of traditionally used test methods, as well as the development of new ones.

## 5. Conclusions

Among the huge number of research and test methods employed in the construction sector, two groups can be distinguished: methods used to reveal the technical truth about materials, elements or building and methods used to simple conformity assessments of products placed on the market. Combining these two purposes (which is often found in standard tests) does not always give the desired results.

Conformity test methods which do not ensure adequate results precision, would be able to increase the assessment risk area so much that the assessment becomes irrational. Use of such methods can also generate undesirable economic, environmental, and social effects because of incorrect assessments. Sustainability in the context of test methods means minimizing such effects.

Development of test methods should be preceded the determination of the aim. The initial material test should be a model of reality while a routine test should only be a model of a particular aspect of reality simple enough to deliver predictable results.

Trueness of the test results in the context of the actual conditions of the building working life does not go hand in hand with the precision of the tests, so these two aspects should be split between initial (trueness) and routine (precision) tests.

## Figures and Tables

**Table 1:** Bond strength values, standard deviations of bond strength results for 15 different adhesives (marked as a–p in the first row) and three laboratories (marked as 1, 2, 3 in the first column). The individual laboratory variations, repeatability and reproducibility variations are given as the coefficient of variation v.

Laboratory	a	b	c	d	e	f	g	h	i	k	l	m	n	o	p
**Bond Strength Results, MPa**															
1	0.022	0.026	0.045	0.043	0.042	0.040	0.048	0.047	0.046	0.060	0.044	0.066	0.062	0.084	0.108
2	0.022	0.054	0.046	0.045	0.044	0.057	0.038	0.047	0.059	0.045	0.071	0.093	0.085	0.083	0.099
3	0.034	0.040	0.051	0.056	0.061	0.054	0.066	0.072	0.070	0.079	0.080	0.089	0.103	0.112	0.113
**Average Bond Strength for Three Laboratories for Particular Adhesives**															
	0.026	0.040	0.047	0.048	0.049	0.050	0.051	0.056	0.058	0.061	0.065	0.083	0.084	0.093	0.106
**The *v* Coefficient of Variation of Bond Strength Results for 15 Different Adhesives and Three Laboratories in %**															
1	48.7	24.5	15.6	17.8	16.6	28.4	8.9	17.1	33.1	16.9	9.1	15.4	33.4 ^1^	18.0	11.5
2	64.6	14.6	53.5	12.7	55.6 ^2^	6.8	9.1	12.2	4.5	54.3	12.4	6.8	4.4	8.3	10.8
3	18.5	23.2	35.6	19.4	11.0	10.9	8.6	8.3	16.1	11.9	5.5	9.9	5.6	16.2	8.9
**Repeatability Expressed as Coefficient of Variation ν for Each Adhesive in %**															
	41.9	19.8	38.4	17.4	30.9	15.3	9.0	12.1	18.9	26.5	9.5	10.4	15.1	15.3	10.4
**Reproducibility Expressed as ν Coefficient of Variation of Each Adhesive in %**															
	45.3	39.0	42.3	21.4	34.5	22.5	28.5	28.4	26.6	35.7	29.6	19.8	27.7	22.1	11.4

^1^ Outlier variance (outlier variance significant at the 99% level of confidence in the Cochran test); ^2^ Questionable variance (outlier variance significant at the 95% level of confidence in the Cochran test).

**Table 2:** Four example-approaches to uncertainty estimation.

Approach	Force *F*, *N* Diameter *D*, mm	Random Dispersion of *σ* Results	Coverage Factor, *k*
I approach	Type B evaluation, based on the standard requirements regarding accuracy: *F*-force measurement accuracy 1%. Rectangular PDF ^1^ D-accuracy of the sample preparation 1 mm. Rectangular PDF	No evaluation. Approach based on the assumption that uncertainty relates to the accuracy of the direct measurements only (*F* and *D*)	*k* = 2 (sometimes inappropriate but often used).
II approach		Type A evaluation based on the current test results. 4 results, *ν* = 3 degrees of freedom.	*k* = *t*_0.95_(*ν*_eff_), where *ν*_eff_—value of *t*_p_ from *t*-distribution with an effective degree of freedom *ν*_eff_ obtained from the Welch-Satterthwaite formula. p-fraction of the distribution
III approach		Type B evaluation, based on historical results (e.g., inter-laboratory tests–reproducibility). Normal PDF.	*k* = 2, based on the assumption that the sR component with normal distribution is dominant.
IV approach		Type A evaluation based on the current test results. Normal PDF	No *k*-factor, 95% coverage intervals obtained by the Monte Carlo method [35]

^1^ Probability density function.

**Table 3:** Bond strength and U uncertainty values assessed with the use of I–IV approaches for three laboratories (1–3) and two materials: adhesive ‘n’ and adhesive ‘o’.

Approach	Adhesive ‘n’			Adhesive ‘o’		
	Lab. 1	Lab. 2	Lab. 3	Lab. 1	Lab. 2	Lab. 3
Results, MPa	0.062	0.085	0.103	0.084	0.083	0.112
**Uncertainties, MPa**						
I approach	0.003	0.004	0.005	0.004	0.004	0.005
II approach	0.066	0.011	0.017	0.048	0.020	0.058
III approach	0.074	0.074	0.074	0.065	0.065	0.066
IV approach	0.046	0.047	0.047	0.039	0.041	0.041

**Table 4:** Decisions about rejection or acceptance of the adhesive used for bonding of insulation material to a wall, depending on the laboratory approach used for uncertainty evaluation and the way of using uncertainty in assessment (based on the example of the “n” adhesive).

Way of Assessment	Approach for Uncertainty Evaluation	Laboratory 1	Laboratory 2	Laboratory 3
Guarded acceptance	I	rejection	acceptance	acceptance
	II	rejection	rejection	acceptance
	III	rejection	rejection	rejection
	IV	rejection	rejection	rejection
Simple acceptance	I, II, III, IV	rejection	acceptance	acceptance
Guarded rejection	I	rejection	acceptance	acceptance
	II	acceptance	acceptance	acceptance
	III	acceptance	acceptance	acceptance
	IV	acceptance	acceptance	acceptance

**Table 5:** Medium bond strengths obtained according to ÖNORM (round stamp) and ETAG 004 (square stamp).

Material	Method 1, Round Stamp, *D* = 50 mm, Medium Bond Strength, MPa	Method 2, Square Stamp *a* = 50 mm Medium Bond Strength, MPa
Adhesive 1	0.046	0.068
Adhesive 2	0.066	0.097
Adhesive 3	0.051	0.085
